# Responsibility of consumers for mining capacity: decomposition analysis of scarcity-weighted metal footprints in the case of Japan

**DOI:** 10.1016/j.isci.2020.102025

**Published:** 2020-12-31

**Authors:** Ryosuke Yokoi, Keisuke Nansai, Kenichi Nakajima, Takuma Watari, Masaharu Motoshita

**Affiliations:** 1 Research Institute of Science for Safety and Sustainability, National Institute of Advanced Industrial Science and Technology (AIST), 16-1 Onogawa, Tsukuba 305-8569, Japan; 2 Center for Material Cycles and Waste Management Research, National Institute for Environmental Studies, 16-2 Onogawa, Tsukuba 305-8506, Japan; 3 ISA, School of Physics, Faculty of Science, The University of Sydney, Sydney, NSW 2006, Australia; 4 Graduate School of Frontier Sciences, The University of Tokyo, 5-1-5 Kashiwanoha, Kashiwa, Chiba 277-8563, Japan

**Keywords:** Earth Sciences, Mining Geology, Environmental Policy, Materials Science, Metals

## Abstract

Metal-consuming countries depend on mining activity in other countries, which may impose potential pressure on sustainable metal supply. This study proposes an approach to analyze the responsibility of consuming countries for mining activities based on the decomposition analysis of scarcity-weighted metal footprints (S-MFs) of Japan. The application results to the Japanese final demand (iron, copper, and nickel) demonstrate the significance of country- and metal-specific conditions in terms of metal footprints and mining capacity in assessing the responsibility of consuming countries. Consuming countries can identify influential factors to reduce their S-MFs based on the decomposition analysis by discriminating the directly controllable and uncontrollable factors for consuming countries, which can help to plan different countermeasures depending on the types of the identified influential factors. The proposed approach supports metal-consuming countries to determine the effective options for reducing the responsibility for the sustainability of metal supply.

## Highlights

• Scarcity-weighted metal footprints (S-MFs) of Japan are analyzed for Fe, Cu, and Ni
• S-MFs demonstrate the consuming country-specific responsibility for mining capacity
• Decomposition analysis can identify key factors for the consumer's responsibility
• The analysis provides the insights on metal supply risks and their alleviation

## Graphical abstract

**Figure:**
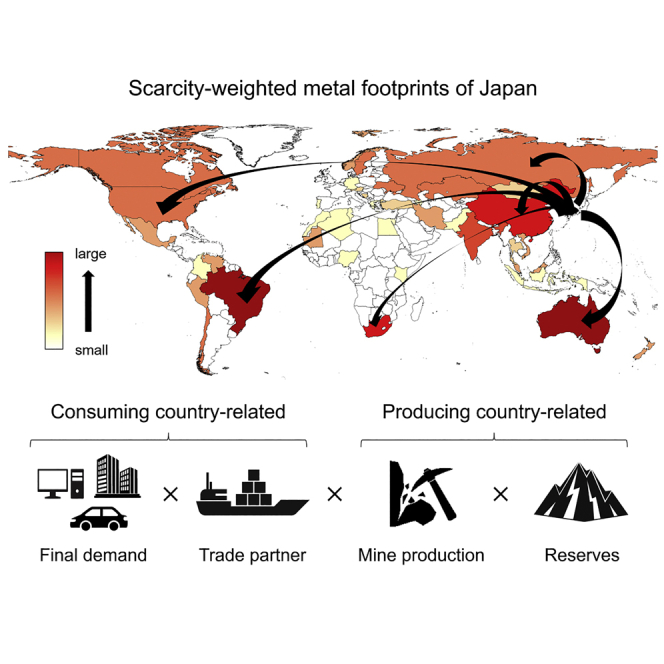

## Introduction

Metals play an essential role in modern human life, and the demand for metals is predicted to increase due to global population and economic growth and the increased adoption of low-carbon technology (Christmann, 2018; Elshkaki et al., 2018; Lee et al., 2020; UNEP, 2013; Watari et al., 2021). Even if metals are not likely to be exhausted on a global scale for the time being (Jowitt et al., 2020), mining capacity is stressed and mining activity may cease on a local scale, which leads to loss of diversity of supply source or need to expand deposits. Supply source diversity is a critical factor for resilient material supply chains, which is addressed in the criticality assessment of metals (Alonso et al., 2007; Graedel and Reck, 2016; Schrijvers et al., 2020; Sprecher et al., 2015). The concept of criticality is generally used to evaluate the probability of supply disruptions (i.e., supply risk) and the vulnerability to supply disruptions for a given material (Achzet and Helbig, 2013; Dewulf et al., 2016; Helbig et al., 2016). As concern for metal criticality has been growing, it is important to use metals so as not to lead to higher criticality in the future. Furthermore, the increasing metal demand may threaten mining capacity on the local scale, which may require access to new or previously uneconomic deposits. The conversion of resources (including uneconomic amounts of metals) to reserves over time in response to the increase in mine production has been observed (Jowitt et al., 2020; Mudd and Jowitt, 2018). However, previous studies have suggested that the development of new and undeveloped deposits may cause environment, social, and governance (ESG) risks in the local sustainability context, thereby resulting in higher supply risks (Jowitt et al., 2020; Lèbre et al., 2019; Northey et al., 2018; Valenta et al., 2019). Given these facts, metal mining in countries with lower mining capacity is concerned about leading to higher supply risks. Therefore, in addition to the assessment of metal availability on a global scale for the long-term, the consideration of mining capacity on the local scale from a short-term perspective is also crucial for the sustainable use of metals.

As mineral deposits are unevenly distributed worldwide, many countries need to rely on mining in other countries through international trade (Galli et al., 2012). Thus, consuming countries (especially high-consumption developed countries) may induce various mining-related issues in producing countries associated with mining activity, such as pressure on mining capacity, which increases the potential risks to local sustainability in mining. Accordingly, consuming countries should recognize the responsibility for mining activity in producing countries and reduce the burdens imposed on producing countries. Such displacement of burdens through international trade is a topic of increasing research interest and is often referred to as “consumption-based accounting” or “footprinting” (Hoekstra and Wiedmann, 2014; Wiedmann and Lenzen, 2018).

Several studies have analyzed the dependency of consuming countries on international trade through consumption-based accounting of materials (material footprint), metal-related risks, and their drivers (e.g., Bruckner et al., 2012; Liu et al., 2020; Nansai et al., 2015; 2017; Wiedmann et al., 2015). Among them, Vivanco et al. (2017) and Wang et al. (2020) shed light on the local conditions of mining capacities in addition to induced mining by consumers for metal and fossil fuel, respectively. Regarding metals, Vivanco et al. (2017) introduced an indicator of metal scarcity expressed by the ratio of mine production to availability and incorporated it with metal footprints (scarcity-weighted metal footprint [S-MF]). The S-MF demonstrates how consuming countries induce pressure on mining capacities of producing countries and consequently, the potential risks to local sustainability in mining. Although S-MF can provide insight into the responsibility of consuming countries for pressure on mining capacity, Vivanco et al. (2017) simply summed up the S-MFs for all metals considered. The regional distribution of metal scarcity differs significantly between metals (Yokoi et al., 2020). Therefore, a simple summation of the scarcity-weighted metal amounts may not be indicative of metal-specific conditions of pressure on metal scarcity in mining capacity. Furthermore, the S-MFs induced by consuming countries are determined based on a combination of factors (e.g., consumer demand for metals, trade partners, and metal scarcity conditions in producing countries; see Transparent methods), making it challenging to plan actions to reduce potential risks represented by the S-MF. Quantitative analysis of the effects of these factors on S-MFs would assist consuming countries in identifying key influencing factors and in implementing initiatives to reduce pressure on producing countries by strategically managing metal use toward lower criticality of metals. However, such an analysis has not been conducted in previous studies.

Here, we focus on Japan as a consuming country. Japan is ranked as the third-largest economy (based on gross domestic product) following the United States of America and China. Japanese economy requires large quantities of various metals, and it relies significantly on the import of metals and metal-based products due to the scarce availability of metals inside the country. Therefore, Japan is among the main countries responsible for induced mining activity in producing countries. Thus, Japan is a suitable subject for a case study analyzing the responsibility for the pressure on mining capacity of producing countries that demonstrates the importance and potential implications of actions by consuming countries toward sustainable metal use.

In this study, we focus on metal scarcity in mining capacity as an influencing factor of metal criticality. This study aims to evaluate the responsibility for pressure on mining capacity of producing countries associated with the final demand of Japan and to identify key factors for consuming countries to reduce their responsibility. We quantify metal mining in producing countries induced by Japanese final demand throughout global supply chains as “induced mine production” (IND) (Nakajima et al., 2019). Then, we assess the S-MFs of Japan for representative metals for different years (2005 and 2011) and adopt a decomposition analysis (Ang, 2005, 2015) to identify factors influencing temporal changes in the responsibility of Japan for sustainability risks in producing countries and opportunities to reduce these risks. For this analysis, we select iron (Fe), copper (Cu), and nickel (Ni) as target metals. These metals are used in large quantities for various applications and are thus vital to the economy (USGS 2020), which means the impacts for the economy are high if their supplies are restricted.

## Results

### Inconsistency between induced mine production and scarcity-weighted mine production

**Figure 1:**
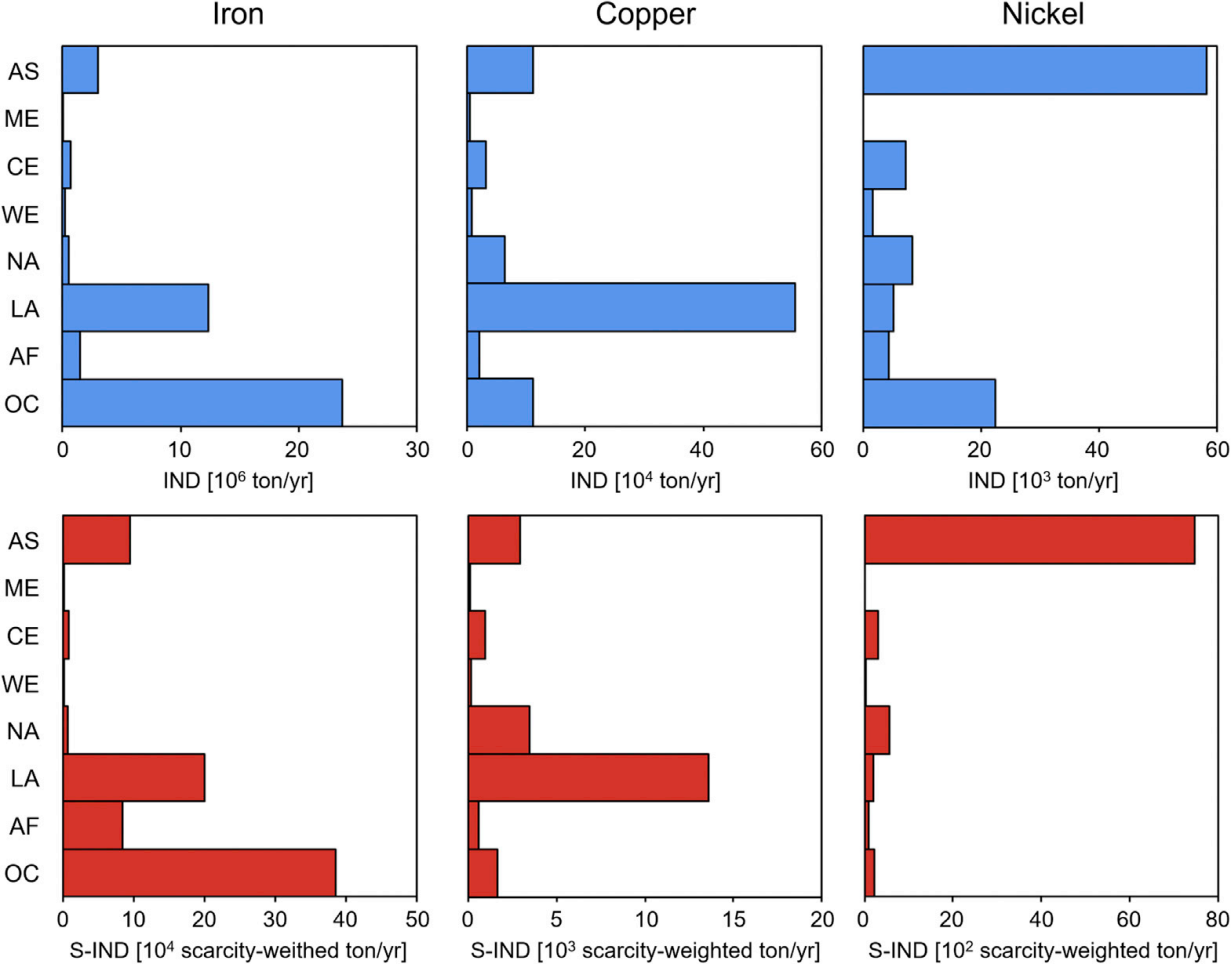
Induced mine production (IND) and scarcity-weighted induced mine production (S-IND) of Japan for Fe, Cu, and Ni in 2011 The upper figures show the amount of mine production induced by Japanese final demand (IND: induced mine production); the lower figures show the induced mine production weighted by country-specific scarcity (S-IND: scarcity-weighted induced mine production). The results of each producing country are aggregated into eight regions. AS: Asia; ME: Middle East; CE: Central Eastern Europe and Russia; WE: Western Europe; NA: North America; LA: Latin America; AF: Africa; OC: Oceania.

**Figure 2:**
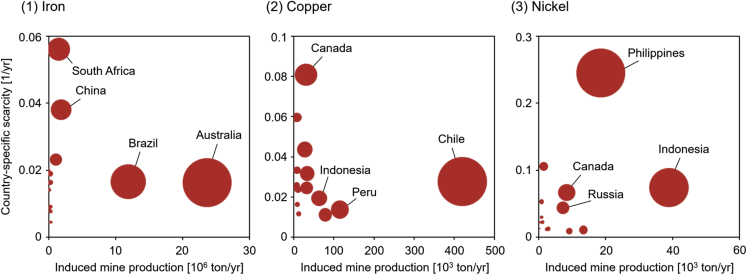
Bubble charts of induced mine production (IND) versus country-specific scarcity (CS) in 2011 The size of the circles represents the scarcity-weighted induced mine production (S-IND) of Japan for each producing country.

IND by consuming countries has different implications depending on the metal scarcity conditions in producing countries. The global distributions of the IND and scarcity-weighted induced mine production (S-IND) of Japan for 2011 are shown in Figure 1 (the detailed results at a country level are available in Figures S1 and S2). The countries and regions on which Japan depends for metal supply differ by metals. Japan largely depends on Oceania (e.g., Australia) and Latin America (e.g., Brazil) for Fe (thus, these regions are the largest contributors to the IND of Japan), whereas Fe sourced from Africa (e.g., South Africa) and Asia (e.g., China) has a greater impact on the S-IND of Japan. The same inconsistency between IND and S-IND is observed for both Cu and Ni. Regarding Cu, the significance of North America is lower than that of Latin America, Oceania, and Asia in terms of IND, whereas North America is relatively more significant than Oceania and Asia in terms of S-IND. The case of Ni is more extreme. The significance of Asia (e.g., Indonesia) for Ni is much greater in terms of S-IND compared with the results for IND. As S-IND is the product of the IND and country-specific scarcity (CS) of a producing country, countries with large INDs and/or high CSs usually show large S-IND values. Figure 2 shows the relationship between IND (horizontal axis) and CS (vertical axis) in each producing country and represents S-IND via the size of a bubble. The countries with large INDs show high significance in S-IND, as do some countries with small INDs but high CS, highlighting the impact of CS on S-IND values. In the case of Fe, Japan induces a large amount of mine production in Australia and Brazil, which results in a large S-IND in both countries. In contrast, the IND of Fe in South Africa and China is relatively minimal; however, the S-IND in South Africa and China cannot be disregarded. If Japan continues to procure Fe from these countries with a relatively high CS, low mining capacity in these countries will increase the ESG risks associated with the development of new and undeveloped deposits. Therefore, the S-IND analysis of Japan at the producing country level can add another relevant aspect of metal supply risk with the analysis of IND. In addition to the direct comparison of the S-IND between different countries, the significance of the S-IND of Japan in a producing country needs to be discussed by comparing it with the total S-IND in a producing country, which is demonstrated in the next section.

### Relative significance of Japanese responsibility for pressure on mining capacity

**Figure 3:**
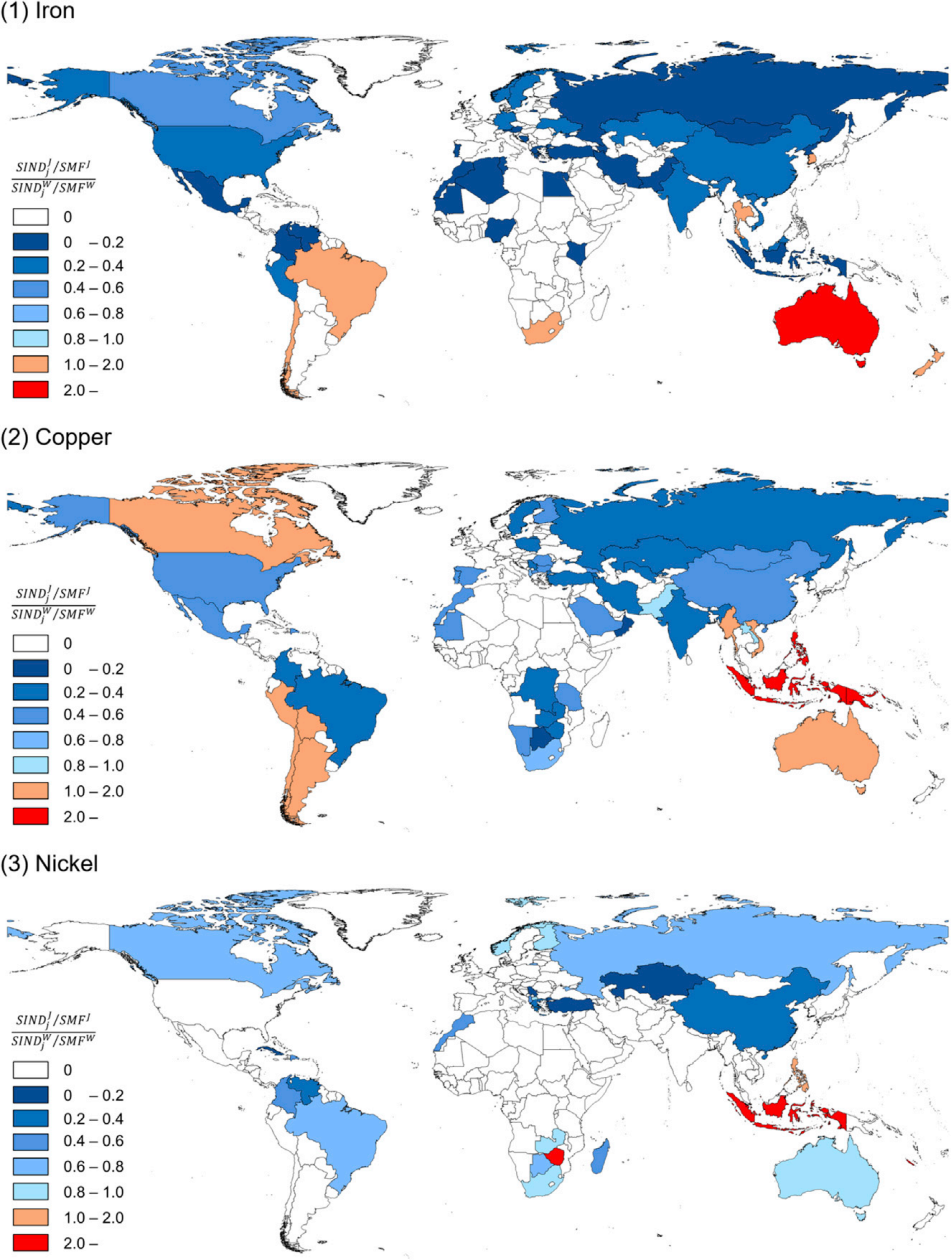
The relative significance of scarcity-weighted induced mine production (S-IND) in each producing country to the scarcity-weighted metal footprint (S-MF) of Japan compared with that of the world total for 2011 If a producing country shows a value larger than 1.0, the relative significance of S-IND in the producing country to the S-MF of Japan is higher than that of the world total. That is, Japan has a higher responsibility for the S-IND in the country compared with the world average.

Consuming countries induce different amounts of mine production in the countries from which they source metals. Therefore, the dependency on producing countries in terms of the S-IND is specific to consuming countries, which can be demonstrated by the share of the S-IND in a producing country to the total S-IND of a consuming country (i.e., S-MF) as the responsibility of a consuming country for a producing country. By comparing the shares of Japan and the world total (all consuming countries), we demonstrate for what countries Japan has relatively higher responsibility compared with other consuming countries. Figure 3 shows the relative significance of Japanese responsibility for the S-IND in producing countries compared with the world total. The relative significance of Japanese responsibility for the S-IND is the highest in Australia, followed by Brazil and South Africa for Fe; Papua New Guinea, Philippines, and Indonesia for Cu; and Indonesia, Zimbabwe, and New Caledonia for Ni. On the other hand, the metal scarcity of these producing countries is different among metals (Figures S3 and S4). Regarding Fe and Cu, the metal scarcity of these countries is fairly low except for South Africa for Fe. On the contrary, scarcity for Ni is relatively high in Indonesia and Philippines compared with that in other producing countries, whereas Japan depends on the Ni extraction in these countries.

The dependency on producing countries and their conditions of metal scarcity affect the relative significance of Japanese responsibility for the S-MF as a total. In the case of Fe and Cu in 2011 (Tables S1 and S2), the Japanese share of the total world S-MF (Fe: 2.91%, Cu: 5.10%) is smaller than that of the IND (Fe: 3.38%, Cu: 5.60%), indicating that Japan induces mine production in producing countries with relatively low scarcity compared with the world average of consuming countries. On the contrary, in the case of Ni (Table S3), the Japanese share of the total world S-MF (6.58%) is larger than that of the IND (5.55%). This is because Japan procures Ni from countries with relatively high scarcity than does the average consuming country. Therefore, the choice of trade partners for metal and metal-containing products supply, which is associated with the composition of producing countries, is an important factor for the S-MF of consuming countries, along with the amount of induced mine production and the scarcity conditions in producing countries.

### Influential factors on the scarcity-weighted metal footprints

**Figure 4:**
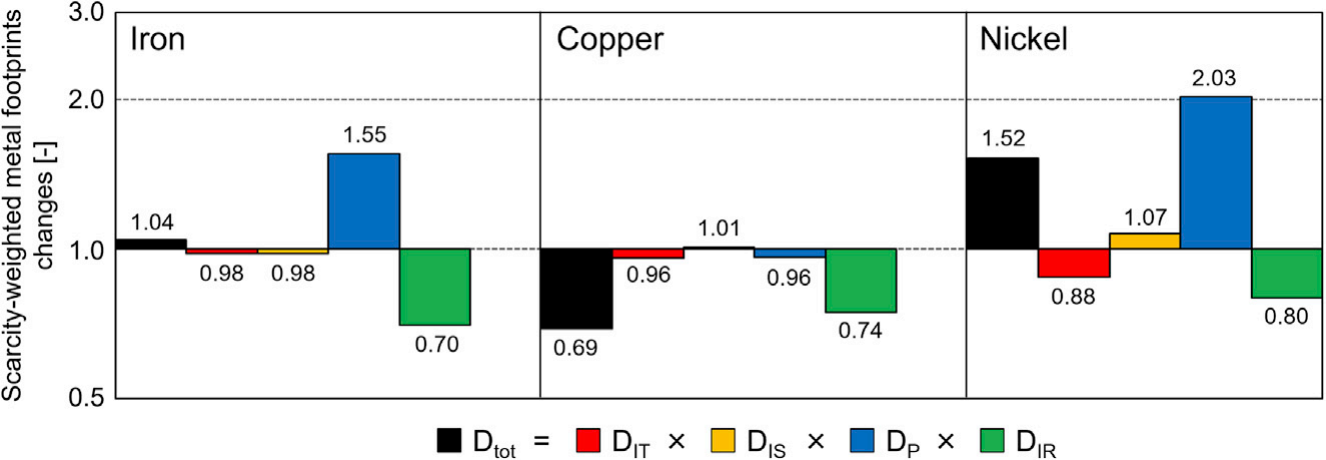
Decomposition analysis of changes in the scarcity-weighted metal footprints of Japan between 2005 and 2011 The vertical axis is displayed in logarithmic scale with base 2. *D*_*tot*_ values (black bar) are the ratios of the scarcity-weighted metal footprints (S-MFs) of Japan in 2005 and 2011. Values for other factors (*D*_*IT*_, *D*_*IS*_, *D*_*P*_, and *D*_*IR*_) represent their contributions to S-MF changes. The product of these values is equal to the ratio of the S-MFs in 2005 and 2011 (*D*_*tot*_). *D*_*tot*_: changes in the scarcity-weighted metal footprints of Japan; *D*_*IT*_: induced mine production effect; *D*_*IS*_: trade partner choice effect; *D*_*P*_: mine production effect; *D*_*IR*_: reserves effect.

The S-MFs of consuming countries are determined based on various factors (induced mine production, choice of trade partners, and scarcity conditions in producing countries) that can temporally vary. Decomposition analysis is effective in identifying factors that influence temporal change in the S-MFs, which can support planning actions to reduce induced pressure on producing countries in terms of strategic management of metals. Figure 4 shows the results of the decomposition analysis of S-MFs changes in Japan between 2005 and 2011 for Fe, Cu, and Ni (results of S-MFs in 2005 are shown in Tables S4, S5, and S6). *D*_*tot*_ values (black bar chart) represent changes in the S-MFs for 2005 and 2011. The values of other factors indicate their contribution to S-MF changes.

The S-MFs (*D*_*tot*_) for Fe and Ni increase in 2011, whereas that for Cu decreases. Regarding the factors that consuming countries can control (*D*_*IT*_ and *D*_*IS*_), the decrease in the IND associated with the final demand of Japan in 2011 contributes to the lower S-MFs for all target metals (induced mine production effect: *D*_*IT*_), whereas trade partner choice effect (*D*_*IS*_) decreases the S-MFs for Fe but increases those for Cu and Ni. The reduction of induced mine production directly leads to decreased S-MF values, whereas the composition of producing countries affects S-MFs more intricately depending on scarcity conditions in producing countries. The decomposition analysis results regarding the trade partner choice effects (*D*_*IS*_) demonstrate whether the choice of trade partner in 2011 is better than that in 2005. However, S-MFs could potentially be reduced further through different choices. To explore the potential for reducing S-MFs by changing the composition of producing countries, the virtual S-MFs (S-MF’) are additionally calculated based on the assumption that Japan induces mine production of these metals in producing countries corresponding to the share of the world average (Table 1). If Japan followed the world average composition of producing countries, the S-MF for all target metals could have been reduced by changing the choice of producing countries in 2005 (Table 1). On the other hand, whereas the Japanese S-MFs for Ni in 2011 could be potentially reduced by following the world average composition of producing countries, those for Fe and Cu in 2011 would increase by shifting the composition of producing countries from Japanese case to the world average case. This indicates that the Japanese composition of producing countries for Fe and Cu in 2011 is better than the world average. On the other hand, the decomposition analysis results (Figure 4) show that the trade partner choice effect (*D*_*IS*_) contributes to the changes in the S-MFs in both directions: reduction for Fe and increase for Cu. Thus, the Japanese composition of producing countries for Cu improves S-MF values in 2011 (relative to the world average; Table 1), whereas the Japanese choice of trade partners for Cu has a further reduction potential of the S-MFs in 2011 compared with 2005.

**Table 1:** Comparison of the actual scarcity-weighted metal footprint (S-MF) and virtual S-MF (S-MF’) of Japan

		Iron	Copper	Nickel
2005	S-MF (scarcity-weighted ton/year)	7.50×10^5^	3.40×10^4^	5.85×10^3^
	S-MF’ (scarcity-weighted ton/year)	6.47×10^5^	3.28×10^4^	5.58×10^3^
	S-MF’/S-MF	0.86	0.96	0.95
2011	S-MF (scarcity-weighted ton/year)	7.81×10^5^	2.35×10^4^	8.90×10^3^
	S-MF’ (scarcity-weighted ton/year)	9.19×10^5^	2.61×10^4^	7.35×10^3^
	S-MF’/S-MF	1.18	1.11	0.83

The S-MF′ is indicative of the S-MF in a case where the share of producing countries of Japan is the same as the world average.

Factors associated with producing countries (*D*_*P*_ and *D*_*IR*_) contribute more significantly to S-MF changes than do consuming country-related factors in all cases for the target metals (Figure 4). For both Fe and Ni, the mine production effect (*D*_*P*_) significantly contributes to increases in S-MF values, although the reserves effect (*D*_*IR*_) decreases S-MFs. For Cu, *D*_*P*_ leads to a slight decrease in S-MFs, whereas *D*_*IR*_ contributes to S-MF decreases more significantly. Both *D*_*P*_ and *D*_*IR*_ are beyond the direct control of consuming countries, whereas it is important for consuming countries to closely observe the situations in producing countries to reduce their responsibility for potential risks associated with mining activity.

## Discussion

In this study, we adopt S-MFs as an indicator representing responsibility of consuming countries for pressure on mining capacity. We demonstrate the inconsistency between mine production and scarcity-weighted mine production of Fe, Cu, and Ni induced in producing countries by Japanese final demand. This implies the integration of the scarcity in mining capacity into metal footprints as the S-MF enables to quantify the potential pressure on mining capacity of metals that cannot be represented by metal footprint indicators (non-weighted by scarcity) adopted in previous studies (e.g., Bruckner et al., 2012; Wiedmann et al., 2015). The scarcity in mining capacity is dependent on conditions in producing countries, and the composition of producing countries is specific to consuming countries. Thus, the responsibility for pressure on mining capacity differs between consuming countries corresponding to the dependency of metal mining on producing countries, as demonstrated by the analysis of the relative significance of the consuming country's responsibility. In addition to the importance of country-specific conditions, the analysis of the S-MFs in this study sheds light on the relevance of metal-specific conditions to the pressure on mining capacity by demonstrating the differences of consuming country's responsibility for pressure on mining capacity among metals. The country-specific scarcity varies more largely for some metals not considered in this study (e.g., Al, Sb, REE, Sn, and W) than for the target metals (Fe, Cu, and Ni) (Yokoi et al., 2020); therefore, metal-specific conditions may be more important for other metals. However, the results of this study indicate the relevance of country- and metal-specific scarcity-weighted footprint analysis in assessing a consuming country's responsibility for pressure on mining capacity of producing countries, an angle not fully investigated in previous studies.

The S-MFs of consuming countries are determined based on multiple factors (e.g., the consumer demand for metals, trade partners, and the conditions of metal scarcity in producing countries). We decompose the S-MFs into four factors and classify these factors as consuming country related or producing country related. The decomposition analysis quantifies the contributions of each factor to S-MF changes for each metal, and the results can support to consider effective options for reducing the responsibility of consuming countries depending on metals. Regarding consuming country-related factors (the induced mine production and the trade partner choice), consuming countries can potentially take direct actions to lower the risks associated with induced mine production via the improvement of resource efficiency and recycling rates, the development of substitute materials, and longer lifetime of final products (Graedel, 2017). When there is more potential to reduce the S-MFs by changing trade partners, understanding the mining capacity conditions in producing countries will support decision-making. For example, a previous analysis of country-specific scarcity of producing countries suggested the potential for substitution of mine production for several metals in some countries with relatively large capacity in reserves at lower pressure from mine production (Yokoi et al., 2020). On the other hand, producing country-related factors (mine production and reserves in producing countries) cannot be directly controlled by consuming countries. In the case of Japan analyzed in this study, producing country-related factors demonstrate a larger contribution to S-MF changes than do the consuming country-related factors for three target metals. However, whereas consuming countries have little power to change producing country-related factors unilaterally, mine production in a producing country is the sum of the mine production induced by consuming countries. Thus, consuming countries can affect mine production in producing countries by recognizing their responsibility of induced mine production for pressure on mining capacity of a producing country. Regarding the reserves effect, consuming countries may contribute to increasing the reserves to alleviate scarcity in mining capacity by supporting mine development. However, as mentioned in previous studies (Jowitt et al., 2020; Valenta et al., 2019), the development of new or previously uneconomic orebodies may cause ESG risks; therefore, it is crucial that development consider local sustainability.

The ESG risks associated with the development of new or previously uneconomic orebodies include the level of political governance in producing countries. The issue of the political governance is recognized as a country risk in criticality assessments, and indicators that represent political risks (e.g., the World Governance Index and the Global Political Risk Index) are adopted to assess the stability of metal supplies (Achzet and Helbig, 2013; Bach et al., 2016; Gemechu et al., 2015). A large S-IND in a producing country (as a part of S-MF) implies an elevated potential for leading to ESG risks associated with the development of new or previously uneconomic orebodies to secure metal supply sources; however, such risks may not occur in all producing countries with large S-INDs. In the case of Japan, Ni-producing countries with large S-INDs demonstrate relatively high political risks, whereas the political risks in Fe- and Cu-producing countries with large S-INDs are relatively low (Figure S5). The stability of metal supply is an important issue in resource securement for governments, industries, and companies; therefore, the S-MFs can contribute to stability assessments by quantifying the potential pressure on the stability of metal supply in terms of the availability of reserves. In these senses, the country risk indicators can complement the S-MFs in terms of the potential ESG risks and provides further insights into the criticality of metals.

Proposed approaches and findings in this study will support consuming countries to realize their responsibility for mining capacity of metals and to manage their supply chains toward sustainable metal use. Nevertheless, there are still multiple tasks that need to be tackled in the future. First, this study focuses on the mine production of three metals (Fe, Cu, and Ni) induced by Japan as a basis for further studies. Analysis of other consuming countries will enable us to understand the performance of each consuming country relative to others, which is expected to contribute to international coordination and resource governance for lower supply risks and sustainable metal use (Ali et al., 2017). In addition, we simply decompose the consuming country-related factors on S-MFs into induced mine production and trade partner choice effects; however, induced mine production could be further decomposed into other factors, such as resource productivity and the usage rate of recovered materials, which describe the performance of society. A detailed analysis of the factors that control demand for metals in consuming countries requires more intensive data collection; incorporating these indicators into decomposition analysis of S-MFs would reveal the performance of consuming countries in detail and provide information to plan more detailed initiatives for improvement. Furthermore, a higher-resolution analysis at the mining site level of producing countries is crucial, as our analysis suggests that producing country-related factors are highly significant for the three target metals. As mining activity varies by mining site, such detailed analysis will provide more useful support for consumer decision-making to reduce the potential risks for mining in producing countries.

### Limitations of the study

This analysis adopts a global link input-output model, which is a hybrid multiregional input-output (IO) model based on Japanese IO tables (Nansai et al., 2009), to estimate induced mine production of Japan. The target years of this analysis are limited to years for which Japanese IO tables are published (2005 and 2011). In addition, data availability and uncertainty are also limitations. Data for mine production and reserves are available from different sources, which are not necessarily completely comparable. Improving data reliability and quantifying data uncertainty are future tasks for data development.

### Resource availability

#### Lead contact

Further information and requests for resources and reagents should be directed to and will be fulfilled by the Lead Contact, Ryosuke Yokoi (r.yokoi@aist.go.jp).

#### Materials availability

This study did not generate new unique reagents.

#### Data and code availability

The data used in this study are available from the corresponding author.

## Methods

All methods can be found in the accompanying Transparent methods supplemental file.

## Supplemental information

Document S1. Transparent methods and Figures S1–S5

Table S1. Induced mine production (IND) and scarcity-weighted induced mine production (S-IND) of Japan and worldwide for Fe in 2011, related to Figure 3

Table S2. Induced mine production (IND) and scarcity-weighted induced mine production (S-IND) of Japan and worldwide for Cu in 2011, related to Figure 3

Table S3. Induced mine production (IND) and scarcity-weighted induced mine production (S-IND) of Japan and worldwide for Ni in 2011, related to Figure 3

Table S4. Induced mine production (IND) and scarcity-weighted induced mine production (S-IND) of Japan and worldwide for Fe in 2005, related to Figure 4

Table S5. Induced mine production (IND) and scarcity-weighted induced mine production (S-IND) of Japan and worldwide for Cu in 2005, related to Figure 4

Table S6. Induced mine production (IND) and scarcity-weighted induced mine production (S-IND) of Japan and worldwide for Ni in 2005, related to Figure 4
