# Supplementary material for: Responsibility of consumers for mining capacity: decomposition analysis of scarcity-weighted metal footprints in the case of Japan
Source: iScience. 2020 Dec 31;24(1):102025. doi: 10.1016/j.isci.2020.102025 (PMC7809504; doi:10.1016/j.isci.2020.102025)
Supplement: Document S1. Transparent methods and Figures S1–S5 [file mmc1.pdf]

iScience, Volume *24*

Supplemental Information

Responsibility of consumers for mining capacity: decomposition analysis of scarcity-weighted metal footprints in the case of Japan

Ryosuke Yokoi, Keisuke Nansai, Kenichi Nakajima, Takuma Watari, and Masaharu Motoshita

Transparent Methods

Metal extraction in each country induced by the Japanese economy

Multiregional input-output (MRIO) models have been adopted to calculate footprint indicators for various environmental problems such as climate change (Hertwich and Peters, 2009), damage to biodiversity (Lenzen et al., 2012), and material extraction (Bruckner et al., 2012; Wiedmann et al., 2015). For the Japanese footprint study, Nansai et al., 2009 developed a global link input-output (GLIO) model, which is a hybrid (mixed unit) MRIO model and specialized for a detailed description of the relationship between the Japanese economy and other countries. Nansai et al., 2015 developed an approach to quantify the metal extraction induced by the Japanese economy by incorporating global metal flows associated with international trade between 231 countries (estimated in Nansai et al., 2014) into the GLIO model. Using this approach, Nakajima et al., 2019 estimated the Fe, Cu, and Ni extraction induced in each country by the Japanese domestic final demand in 2005 and 2011; these estimates were adopted in this study. The GLIO model is based on Japanese input-output (IO) tables, which allows the incorporation of global metal flows with high resolution traded commodity data. The analysis is conducted for 2005 and 2011, as these are the years for which Japanese IO tables were published (which is a limitation of this paper). The metal extraction estimates are expressed not as ore-based weights but as content-based weights. A list and detailed descriptions of the 231 considered countries and regions are available in a previous paper (Nakajima et al., 2019).

Scarcity-weighted metal footprints of Japan

According to van Oers and Guinée, 2016, scarcity describes the state in which "the amount available for use is, or will soon be, insufficient ("demand higher than supply flow")." However, this term is used in different contexts depending on which stage of the supply chain is being considered. When focusing on ore stocks, scarcity is assessed in association with the long-term availability of geological stocks (e.g., extractable global resources); in this context, scarcity is often referred to as "geologic scarcity" (Harmsen et al., 2013; Henckens et al., 2014). On the other hand, scarcity is assessed in the context of supply risk when focusing on the metal user side (Bustamante et al., 2018; Graedel and Erdmann, 2012). This study primarily investigates potential threats to local sustainability in mining induced by Japanese final demand through international trade. Therefore, we focus on the short-term availability of current supply sources and current mining activities. Accordingly, in this paper, "scarcity" is used to represent the ratio between mine production and short-term availability. Here, we adopt the country-specific scarcity indicator using the following equation (Yokoi et al., 2020).

$$CS_{i,j,t} = \frac{P_{i,j,t}}{R_{i,j,t}} \tag{1}$$

where $CS_{i,j,t}$ refers to the country-specific scarcity of metal i in country j for year t (1/yr), $P_{i,j,t}$ refers to the mine production of metal i in country j for year t (ton/yr), and $R_{i,j,t}$ refers to the reserves of metal i in country j for year t (ton). While Vivanco et al., 2017 used reserve base values (which includes reserves, marginal reserves, and subeconomic resources; USGS, 2020) to calculate scarcity indicators within the scarcity-weighted metal footprints (S-MFs), reserves values are used in this paper because we focus on the short-term availability of metals. Reserves are the amount of resources that can be economically extracted at a given time (USGS, 2020) and are influenced by various factors including metal prices and mining technology. Therefore, reserves can fluctuate over time and are not suitable for assessing long-term availability (Calvo et al., 2017; Drielsma et al., 2016a). Nevertheless, reserves data is "direct evidence of current resource availability" and is thus appropriate for the assessment of short-term availability (Drielsma et al., 2016b). The data on mine production and reserves by country are derived from the U.S. Geological Survey (USGS, 2006, 2012).

The S-MFs of Japan are calculated using the following equation (Vivanco et al. 2017).

$$SMF_{i,t}^{J} = \sum_{j} SIND_{i,j,t}^{J} = \sum_{j} \left(IND_{i,j,t} \times CS_{i,j,t} \right) \tag{2}$$

where $SMF_{i,t}^{J}$ refers to the scarcity-weighted metal footprint of Japan for metal i for year t (scarcity-weighted ton/yr), $SIND_{i,j,t}^{J}$ refers to the scarcity-weighted induced mine production (S-IND) of Japan for metal i in country j for year t (scarcity-weighted ton/yr), and $IND_{i,j,t}$ refers to the induced mine production of Japan for metal i in country j for year t (ton/yr), which is estimated by the GLIO model (see the previous section). In addition, worldwide S-MFs ($SMF_{i,t}^{W}$) are also calculated as reference values for comparison with S-MFs of Japan using the following equation.

$$SMF_{i,t}^{W} = \sum_{j} SIND_{i,j,t}^{W} = \sum_{j} \left(P_{i,j,t} \times CS_{i,j,t} \right) \tag{3}$$

Decomposition analysis

To identify factors driving the changes in the S-MF of Japan for each metal between 2005 and 2011, we adopt the logarithmic mean divisia index (LMDI) decomposition approach, which is one of the index decomposition analysis (IDA) approaches (Ang et al., 1998; Ang, 2005). IDA approach is adopted for analyzing the contribution of factors to changes in quantity or intensity indicators, such as

energy consumption, CO_2 emissions, and energy efficiency (Ang, 2015). LMDI approach is a recommended approach among IDA approaches owing to its theoretical foundation, adaptability, ease of use and result interpretation, and perfect decomposition (Ang, 2004). Although it has been mainly adopted in energy consumption and CO_2 emission analyses (Ang, 1995; Ang and Zhang, 2000), it has increasingly been applied in other areas, including material use (Pothen and Schymura, 2015), land requirements (Kastner et al., 2012), and water footprints (Xu et al., 2015).

In this study, the S-MF of Japan in Eq. (2) is rearranged as follows:

$$SMF_{i,t}^{J} = \sum_{j}\left(IND_{i,j,t} \times \frac{P_{i,j,t}}{R_{i,j,t}}\right) = \sum_{j}\left\{IND_{i,tot,t} \times \frac{IND_{i,j,t}}{IND_{i,tot,t}} \times P_{i,j,t} \times \frac{1}{R_{i,j,t}}\right\}$$
$$= \sum_{j}\left(IT_{i,t} \times IS_{i,j,t} \times P_{i,j,t} \times IR_{i,j,t}\right) \tag{4}$$

where $IT_{i,t}$ denotes the total induced mine production by Japan worldwide for metal i and year t (ton/yr), $IS_{i,j,t}$ denotes the share of induced mine production by Japan in country j to the total induced mine production by Japan worldwide for metal i and year t (-), and $IR_{i,j,t}$ denotes the inverse of reserves in country j for metal i and year t (1/ton). $IS_{i,j,t}$ represents the choice of producing countries by Japan. $P_{i,j,t}$ and $IR_{i,j,t}$ are associated with the conditions of local mine production and reserves, which determine the country-specific scarcity. Therefore, the first two parameters ($IT_{i,t}$ and $IS_{i,j,t}$) represent factors that Japan can directly control to reduce the S-MF (referred to as “consuming country-related factors”), while the latter two ($P_{i,j,t}$ and $IR_{i,j,t}$) represent factors that are dependent on conditions in the producing countries that Japan cannot directly control (referred to as “producing country-related factors”).

The LMDI approach is classified into two types of decomposition forms: additive decomposition and multiplicative decomposition (Ang, 2015). In additive decomposition, the arithmetic change of the aggregate indicator is decomposed. In multiplicative decomposition, the ratio change of an aggregate indicator is decomposed. Because the results of these two types of decomposition can be converted to each other, the choice of the decomposition forms depends on the desired presentation and interpretation of the results. In this study, multiplicative decomposition is adopted to analyze the ratio of S-MFs in 2011 to 2005. Based on Eq. (4), changes in the S-MFs of Japan (D_{tot}) are decomposed into four factors: the induced mine production effect (D_{IT}), trade partner choice effect (D_{IS}), mine production effect (D_P), and reserves effect (D_{IR}).

$$D_{tot} = \frac{SMF_{2011}^{J}}{SMF_{2005}^{J}} = D_{IT}D_{IS}D_{P}D_{IR} \tag{5}$$

The contributions of each factor to the change in the S-MF of Japan between 2005 and 2011 are calculated using the following equations (Ang, 2005).

$$D_{IT} = \exp\left(\sum_{j} \frac{\left(SIND_{j,2011}^{J} - SIND_{j,2005}^{J}\right)/\left(\ln SIND_{j,2011}^{J} - \ln SIND_{j,2005}^{J}\right)}{\left(SMF_{2011}^{J} - SMF_{2005}^{J}\right)/\left(\ln SMF_{2011}^{J} - \ln SMF_{2005}^{J}\right)} \ln\left(\frac{IT_{2011}}{IT_{2005}}\right)\right) \tag{6}$$

$$D_{IS} = \exp\left(\sum_{j} \frac{\left(SIND_{j,2011}^{J} - SIND_{j,2005}^{J}\right)/\left(\ln SIND_{j,2011}^{J} - \ln SIND_{j,2005}^{J}\right)}{\left(SMF_{2011}^{J} - SMF_{2005}^{J}\right)/\left(\ln SMF_{2011}^{J} - \ln SMF_{2005}^{J}\right)} \ln\left(\frac{IS_{j,2011}}{IS_{j,2005}}\right)\right) \tag{7}$$

$$D_{P} = \exp\left(\sum_{j} \frac{\left(SIND_{j,2011}^{J} - SIND_{j,2005}^{J}\right)/\left(\ln SIND_{j,2011}^{J} - \ln SIND_{j,2005}^{J}\right)}{\left(SMF_{2011}^{J} - SMF_{2005}^{J}\right)/\left(\ln SMF_{2011}^{J} - \ln SMF_{2005}^{J}\right)} \ln\left(\frac{P_{j,2011}}{P_{j,2005}}\right)\right) \tag{8}$$

$$D_{IR} = \exp\left(\sum_{j} \frac{\left(SIND_{j,2011}^{J} - SIND_{j,2005}^{J}\right)/\left(\ln SIND_{j,2011}^{J} - \ln SIND_{j,2005}^{J}\right)}{\left(SMF_{2011}^{J} - SMF_{2005}^{J}\right)/\left(\ln SMF_{2011}^{J} - \ln SMF_{2005}^{J}\right)} \ln\left(\frac{IR_{j,2011}}{IR_{j,2005}}\right)\right) \tag{9}$$

Additional results

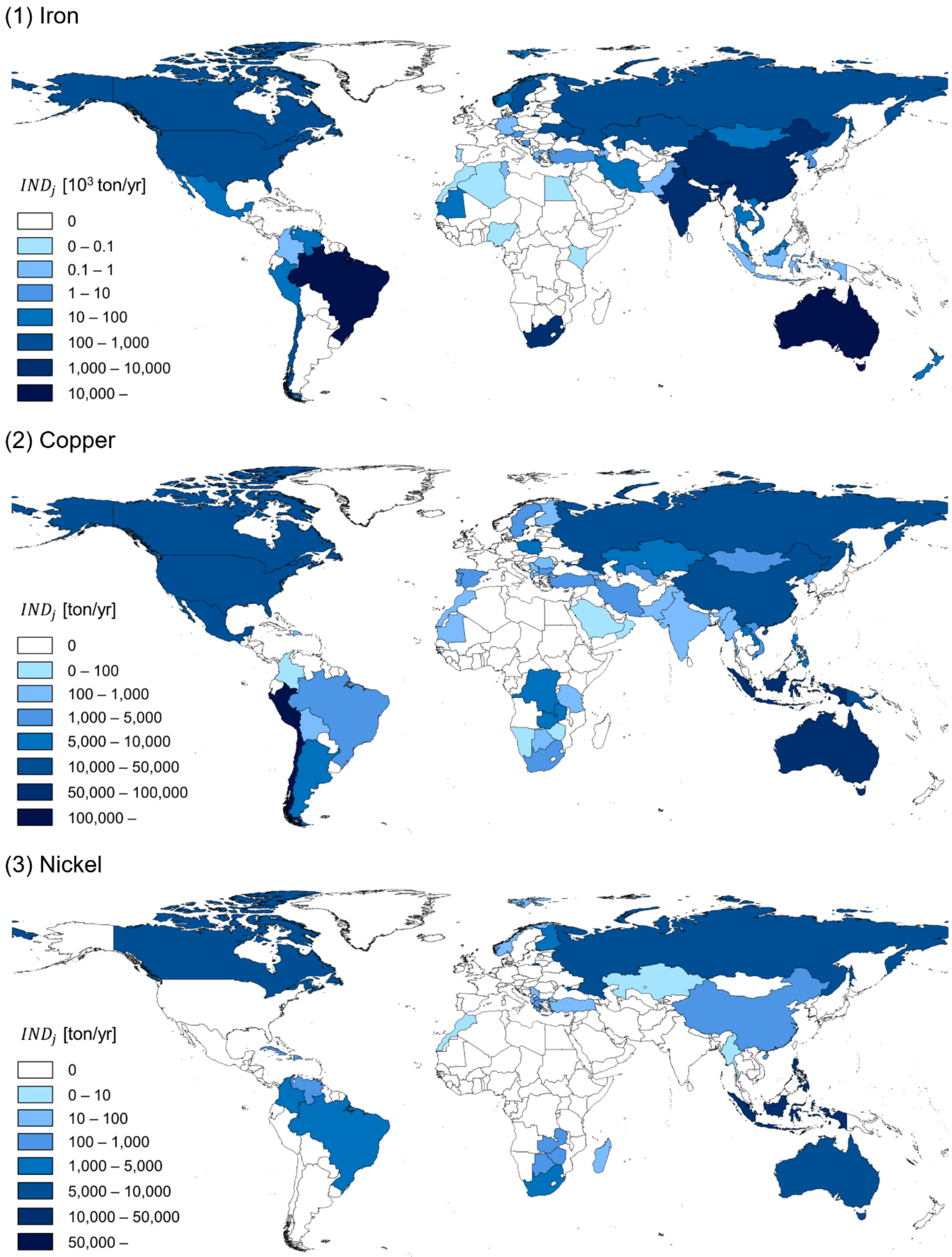

Figure S1. Induced mine production (IND) of Japan for Fe, Cu, and Ni in 2011, Related to Figure 1.

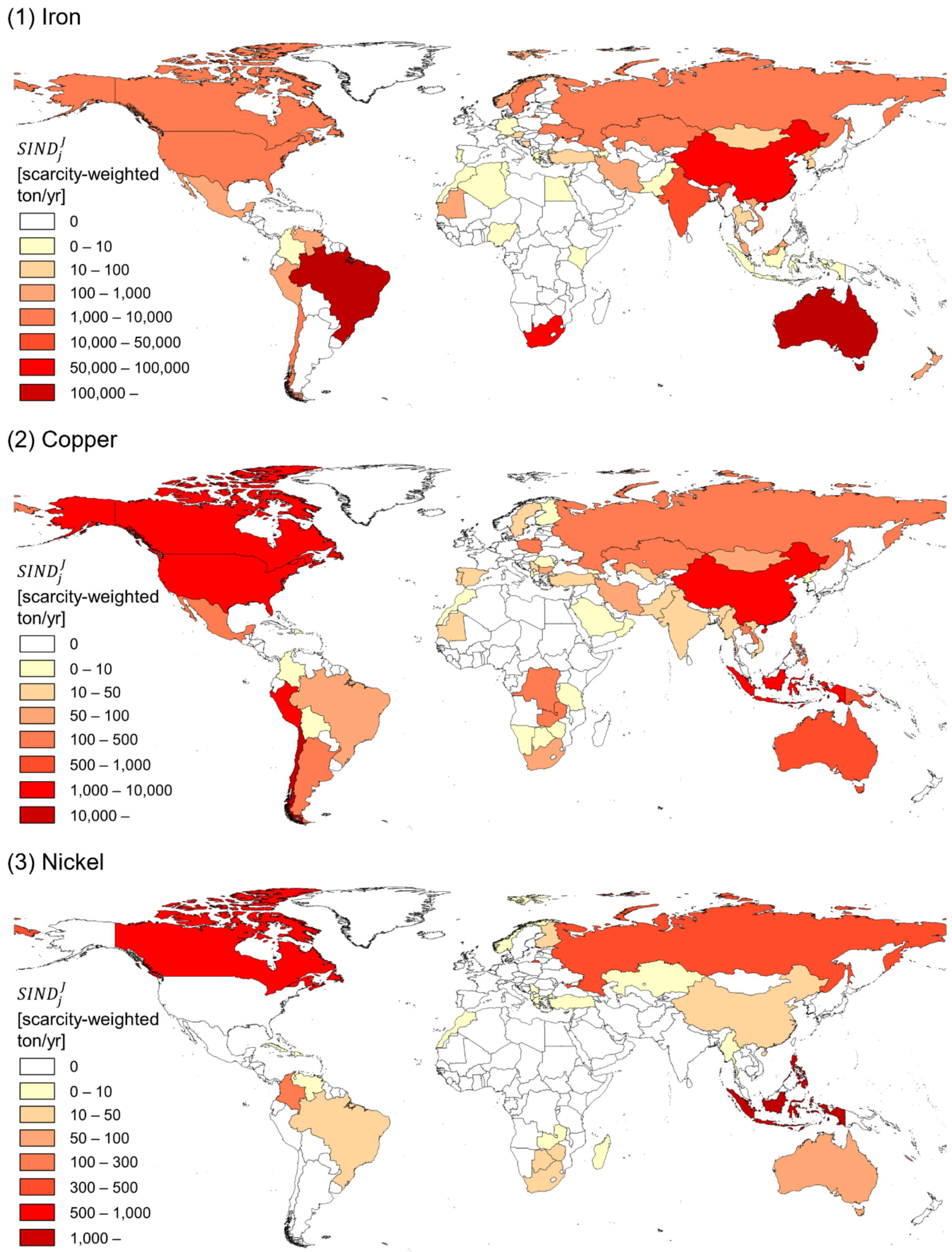

Figure S2. Scarcity-weighted induced mine production (S-IND) of Japan for Fe, Cu, and Ni in 2011, Related to Figure 1.

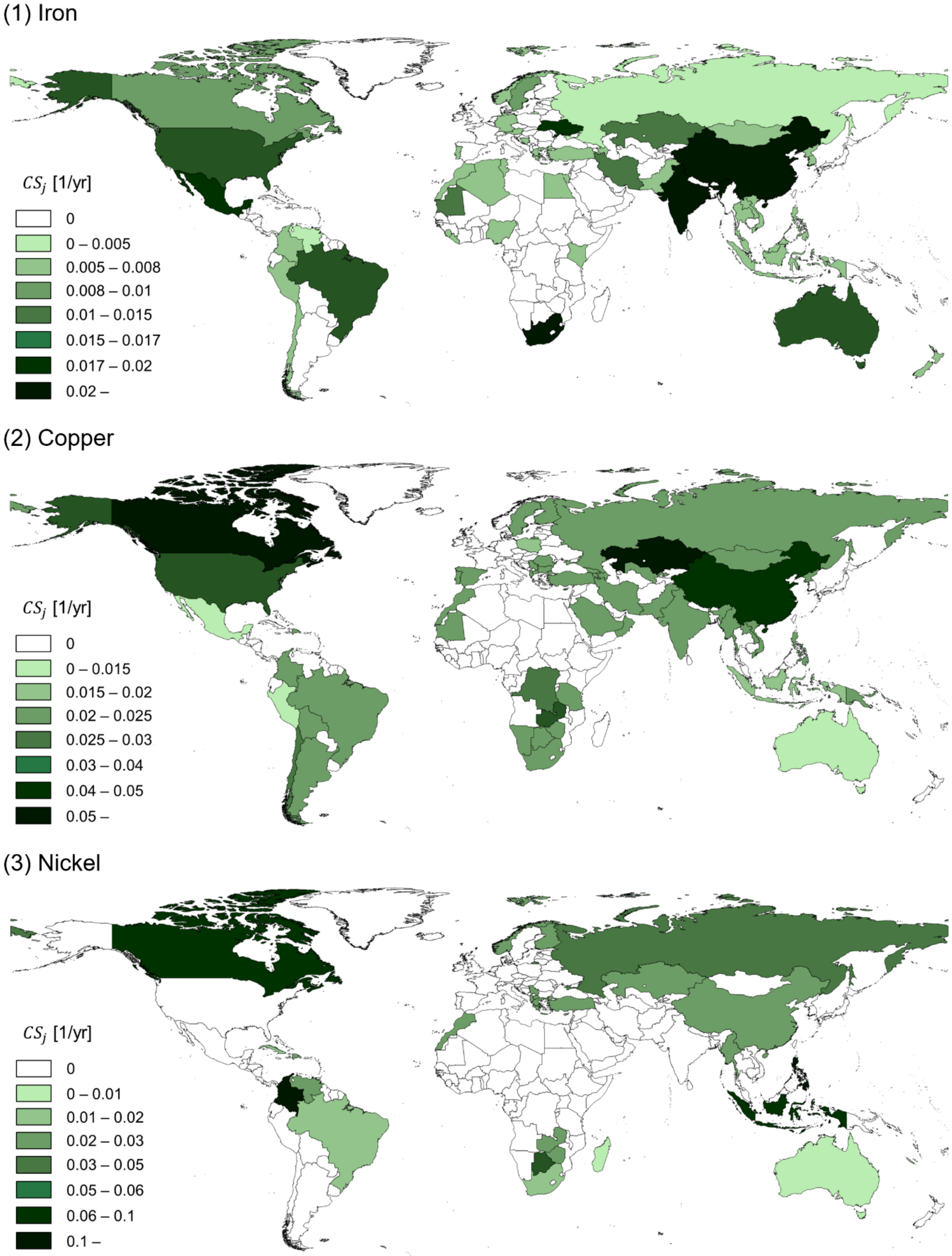

Figure S3. Country-specific scarcity (CS) for Fe, Cu, and Ni in 2011, Related to Figure 3.

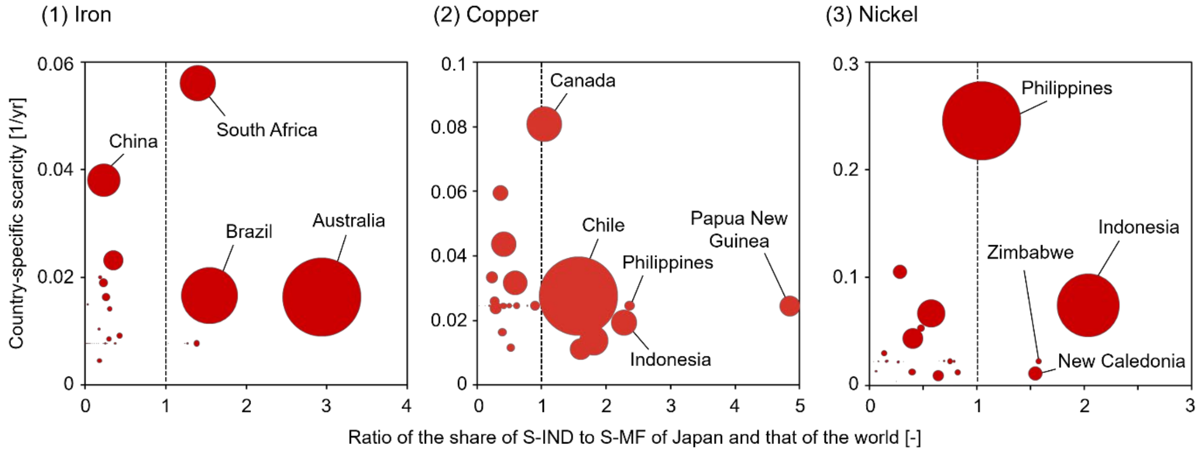

Figure S4. Bubble charts showing the relative significance of Japanese responsibility for metal scarcity in mining capacity and country-specific scarcity in 2011, Related to Figure 3. The size of the circles represents the scarcity-weighted induced mine production (S-IND) of Japan for each producing country.

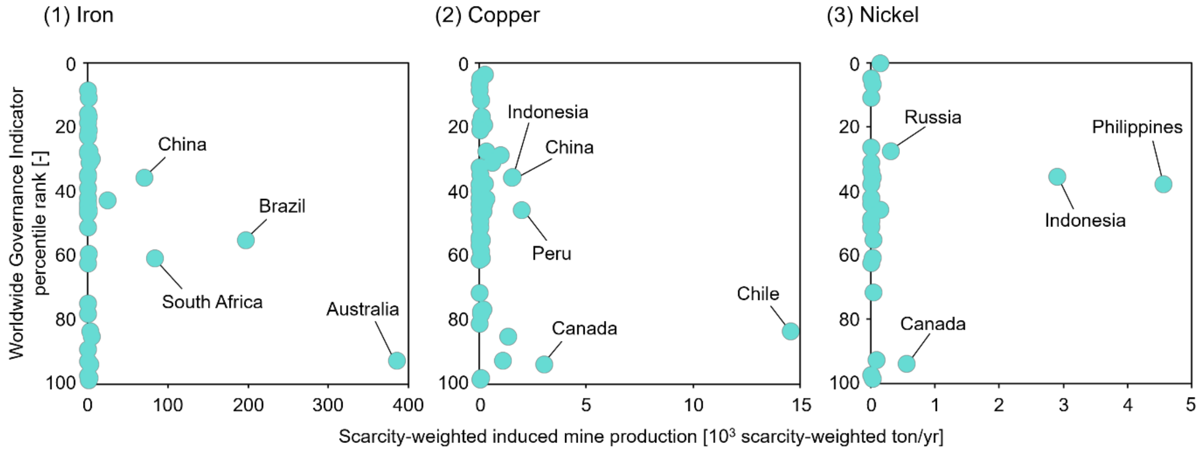

Figure S5. Relationship between the scarcity-weighted induced mine production (S-IND) and political risk in 2011, Related to Figure 1. The horizontal axis represents the scarcity-weighted induced mine production (S-IND) of Japan; the vertical axis represents the World Governance Indicators (WGI) value (Kaufmann et al., 2010). The WGI is published by the World Bank and is comprised of six aspects: voice and accountability, political stability and absence of violence/terrorism, government effectiveness, regulatory quality, rule of law, and control of corruption. In this figure, the average value of the percentile ranks for these six aspects (0–100) is used. A higher WGI value indicates a lower political risk in a country. New Caledonia is excluded from the charts because WGI values have not been calculated for New Caledonia.

References

Ang, B.W. (1995). Decomposition methodology in industrial energy demand analysis. Energy 20, 1081–1095.

Ang, B.W. (2004). Decomposition analysis for policymaking in energy: which is the preferred method? Energy Policy 32, 1131–1139.

Ang, B.W. (2005). The LMDI approach to decomposition analysis: a practical guide. Energy Policy 33, 867–871.

Ang, B.W. (2015). LMDI decomposition approach A guide for implementation. Energy Policy 86, 233–238.

Ang, B.W., Zhang, F.Q., and Choi, K.H. (1998). Factorizing changes in energy and environmental indicators through decomposition. Energy 23, 489–495.

Ang, B.W., and Zhang, F.Q. (2000). A survey of index decomposition analysis in energy and environmental studies. Energy 25, 1149–1176.

Bruckner, M., Giljum, S., Lutz, C., and Wiebe, K.S. (2012). Materials embodied in international trade – Global material extraction and consumption between 1995 and 2005. Glob. Environ. Change 22, 568–576.

Bustamante, M.L., Gaustad, G., and Alonso, E. (2018). Comparative Analysis of Supply Risk-Mitigation Strategies for Critical Byproduct Minerals: A Case Study of Tellurium. Environ. Sci. Technol. 52, 11–21.

Calvo, G., Velero, A., and Valero, A. (2017). Assessing maximum production peak and resource availability of non-fuel mineral resources: Analyzing the influence of extractable global resources. Resour. Conserv. Recycl. 125, 208–217.

Drielsma, J.A., Allington, R., Brady, T., Guinée, J., Hammarstrom, J., Hummen, T., Russell-Vaccari, A., Schneider, L., Sonnemann, G., and Weihed, P. (2016a). Abiotic Raw-Materials in Life Cycle Impact Assessments: An Emerging Consensus across Disciplines. Resources 5, 12.

Drielsma, J.A., Russell-Vaccari, A.J., Drnek, T., Brady, T., Weihed, P., Mistry, M., and Simbor, L.P. (2016b). Mineral resources in life cycle impact assessment – defining the path forward. Int. J. Life Cycle Assess. 21, 85–105.

Graedel, T.E., and Erdmann, L. (2012). Will metal scarcity impede routine industrial use? Mater. Res. Soc. Bull 37, 325–331.

Harmsen, J.H.M., Roes, A.L., and Patel, M.K. (2013). The impact of copper scarcity on the efficiency of 2050 global renewable energy scenarios. Energy 50, 62–73.

Henckens, M.L.C.M., Driessen, P.P.J., and Worrell, E. (2014). Metal Scarcity and sustainability, analyzing the necessity to reduce the extraction of scarce metals. Resour. Conserv. Recycl. 93, 1–8.

Hertwich, E.G., and Peters, G.P. (2009). Carbon footprint of nations: A global, trade-linked analysis. Environ. Sci. Technol. 43, 6414–6420.

Kastner, T., Ibarrola Rivas, M.J., Koch, W., and Nonhebel, S. (2012). Global changes in diets and the consequences for land requirements for food. Proc. Natl. Acad. Sci. 109, 6868–6872.

Kaufmann, D., Kraay, A., and Mastruzzi, M. (2010). The Worldwide Governance Indicators: Methodology

and Analytical Issues. World Bank Policy Research Working Paper No. 5430.

Lenzen, M., Moran, D., Kanemoto, K., Foran, B., Lobefaro, L., and Geschke, A. (2012). International trade drives biodiversity threats in developing nations. Nature, 486, 109–112.

Nakajima, K., Noda, S., Nansai, K., Matsubae, K., Takayanagi, W., and Tomita, M. (2019). Global Distribution of Used and Unused Extracted Materials Induced by Consumption of Iron, Copper, and Nickel. Environ. Sci. Technol. 53, 1555–1563.

Nansai, K., Kagawa, S., Kondo, Y., Suh, S., Inaba, R., and Nakajima, K. (2009). Improving the completeness of product carbon footprints using a global link input-output model: The case study of Japan. Econ. Syst. Res. 21, 267–290.

Nansai, K., Nakajima, K., Kagawa, S., Kondo, Y., Suh, S., Shigetomi, Y., and Oshita, Y. (2014). Global Flows of Critical Metals Necessary for Low-Carbon Technologies: The Case of Neodymium, Cobalt, and Platinum. Environ. Sci. Technol. 48, 1391–1400.

Nansai, K., Nakajima, K., Kagawa, S., Kondo, Y., Shigetomi, Y., and Suh, S. (2015). Global mining risk footprint of critical metals necessary for low-carbon technologies: The case of neodymium, cobalt, and platinum in Japan. Environ. Sci. Technol. 49, 2022–2031.

Pothen, F., and Schymura, M. (2015). Bigger cakes with fewer ingredients A comparison of material use of the world economy. Ecol. Econ. 109, 109–121.

USGS (2006). Mineral Commodity Summaries 2006. https://s3-us-west-2.amazonaws.com/prd-wret/assets/palladium/production/mineral-pubs/mcs/mcs2006.pdf (accessed 20 September 2019).

USGS (2012). Mineral Commodity Summaries 2012. https://s3-us-west-2.amazonaws.com/prd-wret/assets/palladium/production/mineral-pubs/mcs/mcs2012.pdf (accessed 7 July 2020).

USGS (2020). Mineral Commodity Summaries 2020. https://pubs.usgs.gov/periodicals/mcs2020/mcs2020.pdf (accessed 20 September 2019).

van Oers, L., and Guinée, J. (2016). The Abiotic Depletion Potential: Background, Updates, and Future. Resources 5, 16.

Vivanco, D.F., Sprecher, B., and Hertwich, E. (2017). Scarcity-weighted global land and metal footprints. Ecol. Indic. 83, 323–327.

Wiedmann, T.O., Schandl, H., Lenzen, M., Moran, D., Suh, S., West, J., and Kanemoto, K. (2015). The material footprint of nations. Proc. Natl. Acad. Sci. 112, 6271–6276.

Xu, Y., Huang, K., Yu, Y., and Wang, X. (2015). Changes in water footprint of crop production in Beijing from 1978 to 2012: a logarithmic mean Divisia index decomposition analysis. J. Clean. Prod. 87, 180–187.

Yokoi, R., Nansai, K., Hatayama, H., and Motoshita, M. (2020). Significance of country-specific context in metal scarcity assessment from a perspective of short-term mining capacity. Resour. Conserv. Recycl. 105305.
